# Rewiring cell signalling through chimaeric regulatory protein engineering

**DOI:** 10.1042/BST20130138

**Published:** 2013-09-23

**Authors:** Baojun Wang, Mauricio Barahona, Martin Buck, Jörg Schumacher

**Affiliations:** *Department of Mathematics, Imperial College London, London SW7 2AZ, U.K.; †School of Biological Sciences, University of Edinburgh EH9 3JR, U.K.; ‡Department of Life Sciences, Imperial College London, London SW7 2AZ, U.K.

**Keywords:** bacterial enhancer binding protein, cell signalling, chimaeric protein, protein engineering, synthetic biology, two-component signalling system, AAA+, ATPase associated with various cellular activities, bEBP, bacterial enhancer-binding protein, DHP, dihydropyridine, HK, histidine kinase, HTH, helix–turn–helix, N-WASP, neuronal Wiskott–Aldrich syndrome protein, RR, response regulator, TCS, two-component signalling

## Abstract

Bacterial cells continuously sense and respond to their environment using their inherent signalling and gene regulatory networks. Cells are equipped with parallel signalling pathways, which can specifically cope with individual input signals, while interconnectivities between pathways lead to an enhanced complexity of regulatory responses that enable sophisticated adaptation. In principle, any cell signalling pathway may be rewired to respond to non-cognate signals by exchanging and recombining their underlying cognate signalling components. In the present article, we review the engineering strategies and use of chimaeric regulatory proteins in cell signalling pathways, especially the TCS (two-component signalling) system in bacteria, to achieve novel customized signalling or regulatory functions. We envisage that engineered chimaeric regulatory proteins can play an important role to aid both forward and reverse engineering of biological systems for many desired applications.

## Introduction

Most bacteria live in a continuously changing environment. In order to survive, they have evolved many signalling pathways to sense various extra- and intra-cellular signals and then respond accordingly by controlling their cognate downstream gene regulatory networks to adapt to the changes. Proteins involved in cell signalling pathways are typically modular, comprising highly conserved domains and residues. Therefore researchers have engineered variations of these proteins to endow them with customized characteristics. For example, both directed evolution and structure-based rational design approaches have been applied to modify scaffold [1] and regulatory [2] proteins to either increase reaction rates in metabolic pathways or respond to new input signals. However, it is much more challenging to reliably engineer protein-based signalling circuits, as compared with the design of gene regulatory circuits [3–5]. This is largely due to the complex nature of protein folding, the relative unpredictability of tertiary structures, and our limited understanding of protein–protein interfaces. However, the inherent modularity of many allosteric signalling and transcriptional activator proteins suggests that we might be able to alter their regulatory input and actuator output specificities through rational homologous or heterologous domain recombination [6,7]. In the present article, we describe general principles that underlie such signalling pathways and their importance when designing synthetic rewired signalling pathways with discussion of examples where this has been achieved. We also outline future directions and challenges.

## General features and considerations when rewiring TCS and phosphorelay signalling

**Figure 1:**
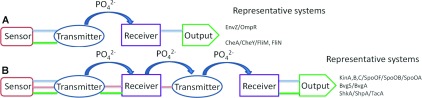
Modularity and covalent domain linkage (horizontal lines indicate covalent linkage) in TCS systems (**A**) and phosphorelays (**B**) Sensor and output domains are versatile in structure and function, whereas transmitters and receivers are highly conserved. (**A**) In TCS systems such as EnvZ/OmpR (blue linkers), the HK comprises a sensor domain and transmitter domain and the RR protein is composed of receiver and output domain. In other TCS systems, the receiver and output functions act *in trans*. (**B**) In more complex phosphorelays, additional receiver transmitter modules are found and may serve to integrate additional inputs into the signalling pathway. The phosphotransfer is directional from transmitter histidine to receiver aspartate to transmitter histidine and so forth. We speculate that the differences in *cis*/*trans* domain linkage in TCS may realize particular signalling requirements and trade-offs with regards to signalling fidelity, network complexity and control stringency (see the text for details).

TCS (two-component signalling) transduction is the most prevalent signalling paradigm that bacterial cells use to sense and respond to the outside world [8]. TCS typically comprises a sensor HK (histidine kinase) and a cognate RR (response regulator) (Figure 1A). The HK is the input sensor receptor, which the cell uses to detect the input signal, for example, small chemical molecules, light, heat or osmolarity. Upon signal perception, the HK autophosphorylates and the phosphoryl group is then transferred through the HK transmitter domain to a conserved aspartate residue within the receiver domain of the RR. The autophosphorylation and phosphotransfer functions reside within the distinct HATPase [histidine kinase/ATPase; also called CA (catalytic and ATP-binding) domain] and HisKA [histidine kinase A; also called DHP (dihydropyridine) domain] domains respectively [9], and we will simply refer to these as the transmitter module. The phosphorylation of the RR generally activates the inherent function of an output domain or module. Most commonly, the output domain comprises a DNA-binding and transcription regulator function, leading to phenotypic changes in motility, growth, morphology, metabolism and so on.

In more complex signalling, additional transmitter and receiver modules are found in phosphorelays, which may serve to integrate additional inputs into the signalling pathway [10]. Importantly, both the transmitter domains and the receiver domains of TCS systems and phosphorelays are remarkably conserved, as is the biochemistry that governs the phosphotransfer between them [11]. In phosphorelays, the second transmitter (Figure 1B) comprises, as far as is known, only the phosphotransfer function of the HisKA domain. This arrangement may ensure directionality of the phosphorelay since the phosphotransfer potential of the donor module must be higher than that of the receptor.

In order to propagate signals, modules of a signalling pathway have to physically interact. Hence binding specificity between modules is a key requirement for signalling fidelity. The specificity between modules is determined by the physicochemical complementarity of their interacting surfaces. Indeed, synthetic rewiring of signalling pathways is challenging because of our limited understanding of how to engineer novel complementary protein or domain surfaces with the desired functionalities. Despite a wealth of available structural data of complementary interfaces between proteins and domains, our knowledge remains largely descriptive and only a few generalizable patterns of protein and domain interfaces exist [12]. Computational tools to predict protein–protein docking that would allow a rational approach to design novel complementary interfaces are being developed and, although still error-prone [13], they may increasingly guide and accelerate the design of functional interfaces between signalling modules.

Another important consideration when designing synthetic signalling pathways is the identification of which is the relevant step of the pathway to be rewired. Figure 1 shows the diversity of protein/domain architectures of representative members, where, in some cases, transmitter, receiver and output domains are sometimes covalently linked to form multi-domain proteins, whereas, in others, they are expressed as orphaned domains. It is reasonable to assume that the evolution of such different arrangements has a bearing on the particular signalling performance and features of the system, such as sensitivity, speed, fidelity, signal amplification, noise and control stringency. *Trans*-acting modules are predicted to require high physicochemical specificity between them in order to avoid unwanted cross-talk between different signalling pathways comprising conserved domains. In *cis*-acting modules, where the components are fixed with one another and in stoichiometric ratios, high specificity may not be required due to the very high local concentrations of the linked modules. However, the covalent link between signalling modules may allow for direct and thus stringent output control. Although this hypothesis has yet to be experimentally proved, it is worth noting that *cis*-acting modules are much more prevalent at the end (receiver-output) of signalling pathways [14], where control stringency may be better achieved through direct domain–domain allosteric control. For instance, the receiver domain of the RR FixJ stringently represses the otherwise strong constitutive transcriptional output domain to tightly control nitrogen fixation in *Sinorhizobium meliloti* [15]. Interestingly, the unusual orphan CheY receiver module (Figure 1A) needs to interact with both FliM and FliN in order to change chemotactic behaviour, a task that may best be achieved through a diffusible CheY module [16].

Although building protein signalling circuits as a means to rewiring cell signalling is far from routine, several encouraging examples have demonstrated the feasibility and promise of synthetic protein-based signalling circuits to program cellular behaviours. To date, various signalling and regulatory proteins have been modified by domain combination, swapping or evolution to render them responsive to novel input signals or able to act on non-cognate target substrates. In the following sections, we discuss the current advances and challenges in this area where different chimaeric regulatory proteins are engineered alongside the various stages of the cell signalling pathways.

## Rewiring cell signalling by swapping the input sensor domain of the sensor kinase

Since the HKs in TCS systems have a modular structure comprising two conserved domains (the sensor and transmitter domains), we can hypothesize that the HK sensor domain can be swapped between different signalling proteins so that the downstream network of a signalling pathway can respond to a non-native input signal.

In a pioneering proof-of-principle bacterial photographing system, a synthetic light-sensitive chimaeric sensor kinase (Cph1–EnvZ) was made in *Escherichia coli* by fusing the photoreceptor domain of the phytochrome Cph1 protein from cyanobacterium *Synechocystis* to the intracellular signal transduction domain of the *E. coli* EnvZ kinase [17], yielding a functional sensor chimaera (Cph8). As a result, the engineered bacteria with a *lacZ* reporter under the RR OmpR controlled promoter can express β-galactosidase to turn the cells black when exposed to red light. Using this chimaeric light sensor kinase, the authors then followed up this work by subsequently creating a bacteria-based dark/light edge detection programme [18].

## Rewiring cell signalling by modifying the transmitter domain of the sensor kinase

Alternatively, swapping or modifying the transmitter domains, the downstream RRs can be activated by an alternative input signal. As a proof-of-concept of this idea, Skerker et al. [19] showed that the specificity of an HK to its RR in TCS can be rewired by rational mutation of key specificity amino acids within and/or transplanting DHP subdomains within the HK domain. They established that the *E. coli* EnvZ kinase can be rationally altered to phosphorylate the non-cognate RRs CpxR and RstA instead of the cognate OmpR *in vitro*. In another study, Xu et al. [20] created NarX–FrzCD chimaeras by coupling the nitrate responsive NarX transmembrane sensor domain to the transmitter domain of the cytoplasmic chemoreceptor FrzCD. The resulting chimaera enabled *Myxococcus xanthus* to chemotactically respond to nitrate, which the wild-type strain cannot respond to, independently of cellular metabolism and physiology. Both of these examples indicate the possibility of rewiring the cell native TCS by rationally designing the signalling kinase to act on a non-cognate RR, thus initiating a reprogrammed signalling response.

## Rewiring cell signalling by swapping the output domain of the RR

**Figure 2:**
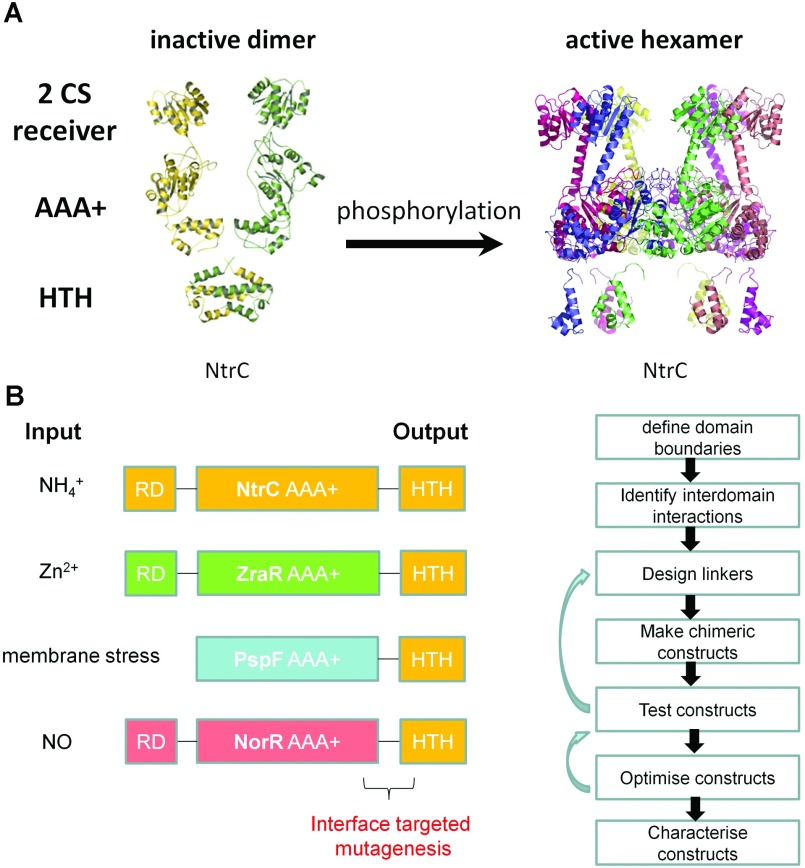
Engineering functional domain exchanged synthetic EBPs (enhancer-binding proteins) to rewire signalling networks (**A**) Structural model of the phosphorylation-induced conformational changes and oligomer assembly of NtrC (adapted with permission from [21,31]). The interfaces between the receiver domain and the AAA+ domain and that between the AAA+ domain and the HTH domain play important regulatory roles in various NtrC homologues through inter-domain allosteric control and determine transcription control stringency [32] (**B**) Engineering strategy for functional domain exchanged synthetic EBPs to rewire signalling networks. Engineered chimaeric EBPs are constructed by coupling the HTH DNA-binding domain of one EBP (e.g. NtrC) to the receiver and AAA+ domains of a non-cognate bEBPs such as ZraR (zinc), PspF (membrane stress), NorR (NO). The functional chimaera can then be selected out by interface-targeted mutagenesis by randomly mutating the linker region between the two coupled domains.

**Figure 3:**
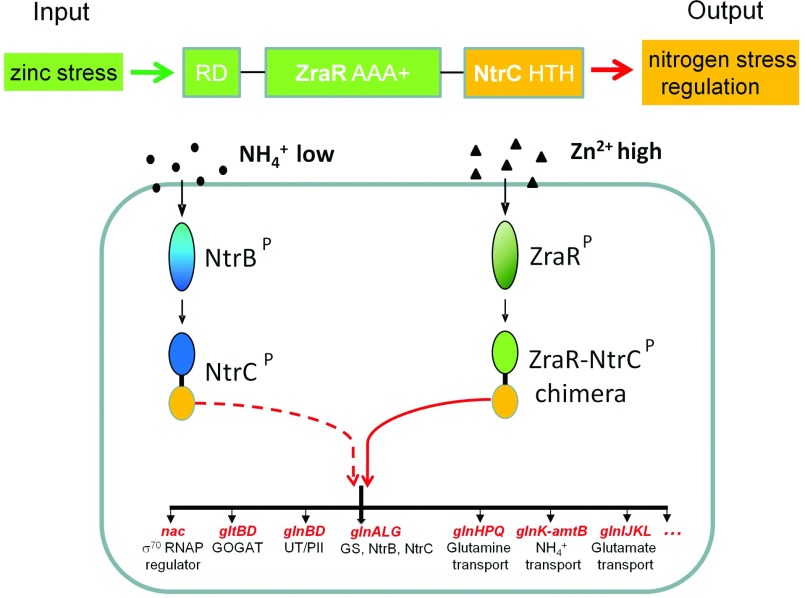
Synthetic systems engineering strategy to rewire the nitrogen regulatory network and enable regulon-wide transcription control NtrC, the central nitrogen stress regulator, directly regulates more than 20 operons of the nitrogen stress-response network. The engineered ZraR–NtrC chimaera can therefore specifically regulate NtrC-targeting promoters under zinc stress signal to control the nitrogen stress transcriptional regulon in *E. coli* Δ*glnG*.

The output domain of a RR is at the end of TCS pathways and is typically a DNA-binding module needed to trigger transcription of downstream genes (Figure 1A). Therefore the output domain largely provides the promoter DNA recognition specificity with a highly modular structure. We have initiated work to engineer receiver/output rewired signalling of TCS systems by exchanging functional domains of bEBPs (bacterial enhancer-binding proteins). Functional receiver/output rewired signalling would complete the proof-of-principle of rewiring TCS between sensor and transmitter [17], transmitter and receiver [19], and receiver and output. Our particular focus is in engineering a synthetic signalling system that allows for stringent output control and for regulon-wide transcription control, instead of single-input single-output signalling systems. Our strategy is outlined in Figures 2 and 3, combining the promoter specificity DNA-binding domain of the master bEBP regulator NtrC with the receiver containing moieties of other bEBP (Figure 2), with the aim to control NtrC-dependent genes with non-cognate signals (Figure 3). Although it would be premature to report on preliminary results, our strategy may illustrate a more general engineering strategy for rewiring signalling at the protein level and its potential for novel cell perturbation tools.

In principle, any two or more domains can be fused by introducing tethering linkers between them. This is readily done and of great value for instance in co-localization studies, by fusing GFP to a protein of interest. However, in signal transduction pathways, specificity between modules is essential for signalling fidelity and control stringencies. In the aforementioned Cph8 chimaera, signalling fidelity downstream of the EnvZ module was provided by the native EnvZ/OmpR intracellular signalling pathway. The functional rewiring relied on exchanging the extracellular sensor moiety in a way that it could still communicate signal perception to the HK domain, where the transmembrane domain must play a structural coupling role across the membrane. The authors constructed a small chimaeric library after defining domain boundaries based on sequence alignments and tested a number of constructs in which the Chp1 moiety length differed while the EnvZ transmembrane moiety was fixed. This was necessary to obtain a functional chimaera, indicating that precise design of the domain architecture underlies rewired signalling.

Box 1 **The use of designed chimaeric proteins**

• Study protein functioning and evolution
  The major challenge in rewiring signalling pathways is to design novel interactions capable of faithfully communicating between modules. The nature of the functional engineered constructs can reveal design principles, i.e. how module specificity is achieved and how it may have evolved. Rewiring the phosphotransfer reaction between transmitter and receiver modules in TCS systems [19] has established that the DHP1 subdomain comprises all determinants required for the specific HK–RR signalling, distinguishing between conserved residues involved in general phosphotransfer and those conveying specificity. The relatively small number of specific amino acid substitutions that were required to rewire signalling in such synthetic circuits suggests that the evolutionary barrier to establish novel network connections in natural systems is relatively small.
• Inform protein engineering approaches
  As the number of synthetically rewired signalling pathways increases, so will our understanding of the principles governing signal transduction pathways, facilitating future approaches. Although every signalling connection is different, the component parts are conserved and the underlying biochemistry is similar, enabling rational design that can be based on first principles.
• Design proteins with bespoke functions
  In the present article, we have only discussed the strategies that are currently employed to generate interoperability between signalling modules, in particular those culminating in gene transcription. Such systems may be more accessible since the engineering approach still comprises an element of trial and error and transcriptional output can be readily selected for. However, the potential to recombine other domain functionalities in novel combinations may apply more generally, for example combining metabolic pathways that are hitherto not connected.
• Perturbation tools that act rapidly, reversibly and are tunable
  Rewiring signalling pathways offers new ways to perturb and study cells. By making new network connections that are not present in the native cell, we can investigate how the cell copes. Such perturbations are conceptually different from conventional gene deletions of transcription factors, where the information flow downstream of the deleted gene is disrupted. Rewired signalling pathways can allow for temporal and tunable [29] transcription activation. Although the phenotypic or system level impact of rewired signalling remains to be elucidated, a more systematic cell signalling rewiring approach has allowed us to reveal global control level hierarchies of signalling pathways [30].
• Allow regulon-wide transcription control
  Our approach aims to rewire signalling that would allow the control of transcription of the extensive nitrogen regulon. Most synthetic biology approaches have focused on changing gene regulatory DNA sequences (mainly promoters) for transcription control. To do so for entire regulons would involve the introduction of many such regulatory elements and also require knowledge of all operons of the regulon. Rewired synthetic transcription factors may therefore represent a more accessible and comprehensive mean for regulon-wide transcription regulation.
• Applicable for metabolic engineering
  One of the challenges in metabolic engineering is the complexity of feedback loops that operate in homoeostasis, impairing efforts to optimize the production of desired natural products. Rewired signalling can uncouple such feedback loops while retaining transcription control of genes involved in the pathways leading to the desired product, providing considerable scope for their application in biotechnology.

bEBPs are modular, typically comprising a C-terminal DNA HTH (helix–turn–helix) binding domain, a central catalytic AAA+ (ATPase associated with various cellular activities) domain and an N-terminal regulatory domain [21] (Figure 2A). Upon phosphorylation of the NtrC receiver domain, activation involves major domain and subunit reorganization from inactive dimers into a highly structured hexameric ring [22]. The HTH and regulatory domain interfaces together with the AAA+ domains regulate assembly of the active hexameric architecture [23]. Guided by structure/function information, sequence alignments and co-variance analysis [24], we defined domain boundaries and potential interdomain interactions and constructed chimaeric bEBPs where domain boundaries varied in length and sequence (Figure 2B). Analogues to the Cph8 chimaera, transcription output of chimaeric bEBPs are highly dependent on the length and sequence of the linker residues that are predicted to form a communicating interface between the recombined domains.

Rewiring the signalling of TCS systems through functional domain recombination has provided proof-of-principle that reliable use of protein engineering can open new synthetic avenues to perturb and redirect cellular information flow. It may be argued that the technically simpler exchange of promoter regulatory elements or coding sequences which are the basis of most synthetic biology approaches achieve this objective more readily. However, it has already been underlined that signal rewiring at the protein level is predicted to be faster and less susceptible to noise because it does not require the intermediate step of gene expression to perform a desired cellular function [25]. In addition, a specific advantage of rewiring signalling that controls transcription activation events is the potential of regulon-wide transcription control. Master regulators such as NtrC control many operons whose genes are functionally linked to perform more complex cellular tasks *in vivo* (Figure 3).

## Rewiring cell signalling in other more complex signalling pathways

So far, we have mainly discussed the various strategies for rewiring cell signalling transduction pathways in prokaryotic systems. However, encouraging progress is also seen in reprogramming advanced eukaryotic signalling pathways through engineered membrane-associated or intracellular chimaeric signalling proteins. Below we summarize recent work in this area and outline the employed protein engineering strategies.

In one study, Dueber et al. [7] constructed a synthetic signalling protein to integrate two non-native physiological signals through modular domain recombination and reorganization of the allosteric actin regulatory protein N-WASP (neuronal Wiskott–Aldrich syndrome protein). The researchers engineered variants of N-WASP by combing two heterologous autoinhibitory input domains [PDZ and SH3 (Src homology 3)] with the output domain of N-WASP. The autoinhibition can be released by two competing ligand peptides cognate to the two input domains so as to trigger actin polymerization in *Xenopus* oocyte extract. By varying the positions of the two input domains, as well as their intramolecular ligand-binding affinities, different output gating behaviours were obtained. Using a similar modular domain recombination approach, Yeh et al. [26] generated synthetic guanine-nucleotide-exchange factors that are activated by non-native physiological inputs to rewire cellular morphology pathways in a human cell line. Both of these examples show that simple modular domain recombination can generate functional synthetic protein switches to rewire cell signalling and how a modular framework can facilitate the engineering of such protein switches.

In another study, Bashor et al. [27] constructed a synthetic scaffold protein device to systematically reshape response dynamics of the yeast mating MAPK (mitogen-activated protein kinase) pathway. The Ste5 scaffold protein in the pathway was engineered with an artificial leucine zipper-binding domain to be able to recruit a positive or negative pathway modulator to the native signal transduction process. With this synthetic feedback loop, diverse signalling dynamics such as ultrasensitive, accelerated and delayed mating responses were observed using different combinations of feedback strengths and binding decoys. A synthetic light-controlled reversible protein–protein interaction device was also engineered from the phytochrome signalling network of *Arabidopsis thaliana*, in which the chimaeric signalling protein was able to recruit cytoskeleton-related proteins to the cellular membrane and thus reshape cell morphology spatiotemporally [28].

## Prospects

Synthetic chimaeric proteins hold great promise to rewire cell signalling as a step towards the engineering of designer organisms for bespoke functions. Owing to the modular nature of signalling pathways, the introduction of new (or the swapping of existing) functional modules is an attractive possibility, in accordance with the conceptual approach in synthetic biology to rationally engineer systems through the assembly of predefined components. Such approaches are also attractive because the majority of proteins in Nature have evolved through the recombination of genetic elements that code for functional protein modules [29]. However, the interacting modules within a signalling pathway have evolved to optimize the particular signalling requirements inherent to the system, including the physicochemical complementarities between modules. As the above examples illustrate, rewiring signalling pathways through domain swapping requires careful design and most often some element of trial and error experimentation. Nonetheless, prior knowledge of the system and its component parts have greatly contributed to the successful engineering of synthetic signalling pathways, enhancing our prospects to engineer such systems rationally. Although there is currently no ‘how to do manual’ and each signalling system is different, existing biochemical, structural and molecular biology information can be combined to guide the initial design. In the future, bioinformatics may play an increasing role in the design process, in particular where there is limited experimental data. Although the field is still in its infancy, the examples above showcase the potential usefulness of synthetic regulatory proteins for fundamental science and applied research (Box 1).
